# ADV6209 for Premedication in Pediatric Anesthesia: A Double-Blinded, Randomized Controlled Trial

**DOI:** 10.3390/pharmaceutics14102062

**Published:** 2022-09-27

**Authors:** Markus Zadrazil, Peter Marhofer, Werner Schmid, Daniela Marhofer, Philipp Opfermann

**Affiliations:** 1 Department of Anesthesia, General Intensive Care Medicine and Pain Therapy, Medical University of Vienna, Spitalgasse 23, 1090 Vienna, Austria; 2 Department of Anesthesia and Intensive Care Medicine, Orthopedic Hospital Vienna, Speisinger Strasse 109, 1130 Vienna, Austria; 3 Department of Special Anesthesia and Pain Medicine, Medical University of Vienna, Spitalgasse 23, 1090 Vienna, Austria

**Keywords:** anesthesia, child, premedication, midazolam, pharmacology

## Abstract

ADV6209, a new formulation of midazolam with the addition of γ-cyclodextrin for oral use, has recently been licensed as the first pediatric sedative in the European Union. We compared the clinical efficacy of ADV6209 to the standard formulation of midazolam in premedication to reduce anxiety in children before anesthesia induction in a randomized, double-blinded controlled trial. Eighty children (ASA I/II; age: 2–8 years) scheduled for elective surgery were randomized to receive 0.25 mg kg^−1^ of either conventional midazolam or ADV6209. Assessment tools included the modified Yale Preoperative Anxiety Scale-Short Form (mYPAS-SF) as well as scores for oral acceptance of the premedication and facemask acceptance during inhalational anesthesia induction. Mann–Whitney U and Pearson’s chi-square tests were used for comparisons of outcome parameters. The primary outcome parameter of the study (mYPAS-SF anxiety score 30 min after the drug administration) did not reveal any significant intergroup difference between the ADV6209 group and the conventional midazolam group. Both drugs revealed their efficacy in reducing anxiety and in providing adequate sedation. The premedication dose was accepted by all children in the ADV6209 but rejected by 15% in the conventional midazolam group (*p* = 0.037). Acceptance of facemask placement was not found to differ significantly. No adverse events related to the study medications were noted. ADV6209 was better orally accepted than the conventional midazolam preparation and proved its efficacy in reducing preoperative anxiety. This clinically interesting preparation may alleviate the premedication process of 2−8 year-old children and obviates off-label drug use.

## 1. Introduction

Premedication administered before surgery to reduce stress and facilitate the induction of anesthesia plays an important role in the perioperative management of children [1,2]. Midazolam with its sedative [3], anxiolytic [1] and amnesic [4] effects is a popular drug for these applications and has shown its efficacy in reducing anxiety and in providing adequate sedation [3]. Strictly speaking, however, midazolam is an intravenous formulation. Thus, any oral (or nasal or rectal) administration represents an off-label use, compounded by a lack of acceptance by children, as even fruit juices cannot neutralize its inherently bitter taste.

In 2009, Marçon and colleagues reported on the development of a midazolam solution for oral use that contained γ-cyclodextrin [5]. More recently, the same authors presented similar pharmacodynamic data for this new formulation (ADV6209, Ozalin^®^; Primex, Zug, Switzerland) as for extemporaneous oral solutions of midazolam (Dormicum^®^; Hoffmann-La Roche, Basel, Switzerland) [6]. It is also important to note that ADV6209 has been licensed by EU regulatory authorities as the first pediatric sedative in the European Union.

Thus, ADV6209 holds the promise of eliminating any unregistered and off-label use of the intravenous formulation for oral premedication in children. These considerations prompted us to design a randomized controlled study, using a double-blinded approach in a single tertiary-care center, to assess the efficacy of ADV6209 with regard to patient acceptance, preoperative anxiety, and quality of inhalational anesthesia induction in children. Our null hypothesis was that ADV6209 used for premedication would emerge as superior to conventional midazolam administered in equivalent doses. Our hypothesis was based on the assumption that the better taste of ADV6209 would cause an improved oral acceptance and therefore an enhanced clinical efficacy in terms of an improved anxiolysis. The objective was to evaluate the use of ADV6209, a new oral midazolam solution, for moderate sedation in children, and to compare its sedative effect to conventional oral midazolam.

## 2. Materials and Methods

### 2.1. Study Preparation and Patient Enrolment

Prior to any patient enrolment, we obtained approval of the study protocol from the institutional review board (ethics committee) at the Medical University of Vienna (ref. 1449/2019, approved on 23 July 2019) and registered the study with the Austrian Agency for Health and Food Safety (EudraCT no. 2019-001853-25) and in the ClinicalTrials.gov Register (NCT03931057). From 16 November 2020 to 17 September 2021, we enrolled 80 children (ASA class I or II) aged 2−8 years scheduled for elective surgery at the Division of Pediatric Surgery (Medical University of Vienna). The age group of 2–8 years has been selected because the used questionnaire—the modified Yale Preoperative Anxiety Scale-Short Form (mYPAS-SF) [7,8]—is best validated for children aged two years and older [7,9]. As children older than eight years are not premedicated routinely at our department, we have chosen this age limit. All parents or legal guardians were informed about the study objective as well as the procedures and risks involved and had to give their written informed consent prior to study inclusion. Criteria for exclusion from the study were allergies or intolerance against one of the drugs or drug components to be used in the study. Additionally, participation in the study was regarded as contraindicated given a history of brain injury or mental impairment.

### 2.2. Patient Management and Clinical Study Context

All children were required to comply with our departmental standard for preoperative fasting (6 h for solid food, 4 h for breast and formula milk, 1 h for clear fluids). The premedication under study was administered orally with 0.25 mg kg^−1^ conventional midazolam (0.05 mL kg^−1^) [10,11] in 0.075 mL kg^−1^ syrup with an orange taste, or with 0.25 mg kg^−1^ ADV6209 (0.125 mL kg^−1^). The dosage regimen of oral midazolam in our study is described as satisfactory [10,11] and is recommended by the summary of product characteristics of Ozalin^®^ (ADV6209). After the premedication, the children were under ward in the recovery room and monitored according to our departmental standard. Thirty minutes later, anesthesia was induced via facemask and sevoflurane (8 vol%, FiO_2_ 50% oxygen/air). Parents were not present during the anesthesia induction in accordance with our departmental routine. Standard cardiorespiratory monitoring was performed (electrocardiography, non-invasive arterial blood pressure, peripheral oxygen saturation). The anesthetic procedure matched our daily clinical routine and did not include any study-related extra steps. Postoperatively, the children were transferred to the recovery room and observed for 2 h before being discharged to the ward.

### 2.3. Randomized Group Allocation and Double-Blinding

The children were randomly assigned to a control (off-label conventional midazolam) or a study (on-label ADV6209) group of oral premedication. Balanced groups were ensured via stratification by gender (female/male) and age (2–4|5–6|7–8 years), with half of the patients in each stratum being randomized to the control and the other half to the study group. A person not otherwise involved in the study used a web-based tool (meduniwien.ac.at/randomizer) for randomization. The randomizer provided a randomization number, which was used for identification of the sealed envelopes containing the treatment allocation. The sealed sets of envelopes (one identical looking set serving as backup) containing details on treatment allocation were prepared per patient and kept at a safe place throughout the study. One envelope was opened per patient after randomization and the study drug was prepared according to the treatment allocation. Masking of the group allocation to both the patients and any individuals involved in data collection or treatment (e.g., anesthetists, surgeons) was ensured by both study drugs bearing a close resemblance in taste (orange), appearance (orange) and viscosity as well as by administration of the drugs in identically looking syringes pasted over with a non-transparent tape.

### 2.4. Outcome Measures and Principal Hypothesis

The primary endpoint of the study was patient anxiety 30 min after administration of premedication, immediately before anesthesia induction, which was assessed as laid out in the modified Yale Preoperative Anxiety Scale-Short Form (mYPAS-SF) [8] based on four modes of behavioral expression observed in the children. A detailed description of this scale is provided in Figure S1. Our main hypothesis was that, based on mYPAS-SF scores obtained 30 min after premedication with an equivalent dose of either formulation of the drug, the clinical effectiveness of ADV6209 would emerge from the study as superior to conventional midazolam. Secondary endpoints were defined as patient acceptance both of the premedication administered and of the facemask placed for inhalational anesthesia induction.

### 2.5. Assessment Tools for the Outcome Measures

Anesthesiologists not otherwise involved in the study assessed all scores. Acceptance of the premedication was rated on a four-point scale indicating that the administered dose was accepted readily (=1), with facial grimacing (=2), with vocal disapproval (=3) or rejected/spat out (=4). Preoperative anxiety was evaluated by applying the aforementioned mYPAS-SF tool both before administering the dose and, 30 min later in the operating theatre, right before mask induction. At this point, acceptance of the facemask was rated, again on a four-point scale indicating that its placement was accepted immediately (=1), with mild resistance (=2), with a moderate struggle (=3) or with moderate force required to overcome the resistance (=4).

### 2.6. Literature Search for Documented Effect Sizes, Sample-Size Calculation and Intention to Treat

We performed a literature search of PubMed and Google Scholar to examine the effect sizes of mYPAS score differences within the literature. Studies that had both a control and an intervention group receiving a treatment for preoperative anxiety and used the mYPAS as outcome measure were used in this context. The effect sizes of Cohen et al. [12] in comparable studies dealing with midazolam premedication had a range from 0.48 to 0.65 suggesting a moderate treatment effect [13,14]. The sample sizes described in these studies ranged from *n* = 50 to study *n* = 197 participants [13,14]. We assumed at least a moderate effect size of 0.5 Cohens d for our primary outcome parameter mYPAS-SF 30 min after premedication. Our power analysis revealed that 72 patients are required to have an 80% chance of detecting, as significant at the 5% level, a decrease in the primary outcome measure from 36 in the control group to 30 mYPAS-SF points in the experimental group (SD of outcome 9). We calculated the power analysis with a dropout rate of 2%. The parameters used for the power analysis are summarized in Table S1 [13,14]. Analysis was based on the intention-to-treat principle to account for noncompliance, protocol deviations, withdrawal or indeed any unforeseen events after randomization.

### 2.7. Statistical Analysis

A Kolmogorov–Smirnov test was used to check for normal distribution, followed by a non-parametric Mann–Whitney U-test for intergroup comparisons of metric and not normally distributed data, namely mYPAS-SF scores at baseline (i.e., before premedication) and 30 min later (i.e., immediately before mask induction). Absolute differences between both of these points in time was further categorized as (1) unchanged scores indicating no effect, (2) higher scores indicating an unfavorable effect and (3) lower scores indicating a favorable effect on anxiety. For intergroup comparisons of proportions, we used cross-tabulation and the Pearson’s chi-square test. The chances of false-positive results (type I errors) from multiple testing were reduced by Bonferroni correction, results expressed as medians with interquartile ranges (IQRs) and/or absolute values with percentages and differences considered significant at *p* < 0.05. All operations were performed with IBM^®^ SPSS^®^ statistical software (v. 26.0.0.0; IBM, Armonk, NY, USA).

## 3. Results

**Figure 1:**
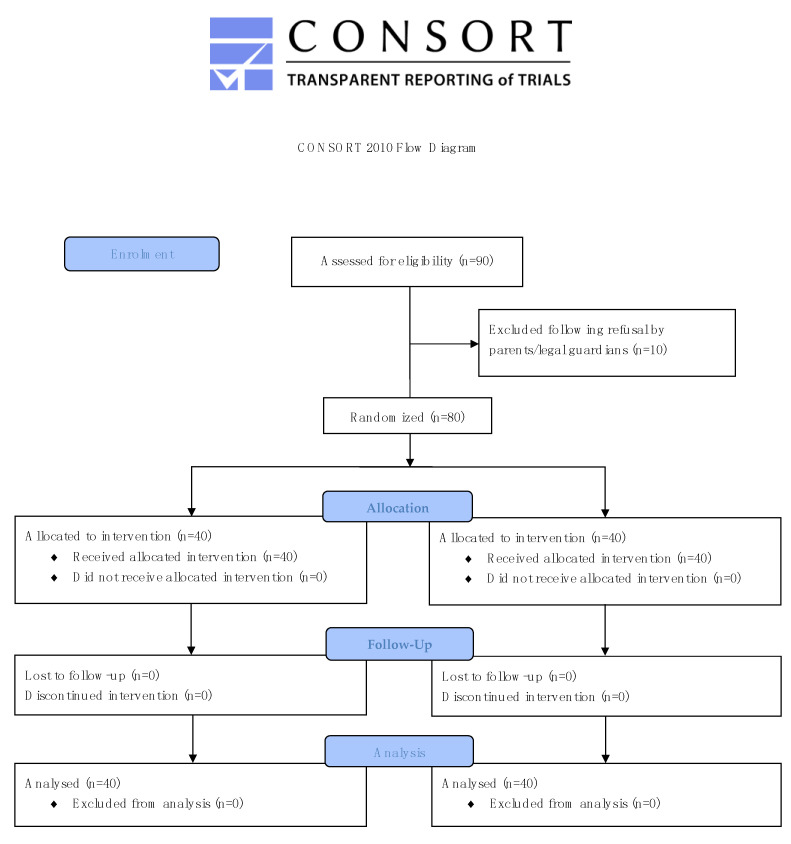
Flow chart of the randomized controlled trial according to CONSORT.

As required by our aforementioned sample-size calculation, we included and were able to evaluate 80 patients, equally randomized to oral premedication either ‘off-label’ with conventional midazolam in the control group (*n* = 40) or ‘as labelled’ with ADV6209 in the study group (*n* = 40). Figure 1 presents the study roadmap in a CONSORT flow diagram as applicable to randomized controlled trials.

Table 1 summarizes relevant patient data. No adverse events related to the study medications were observed.

Table 2 summarizes the mYPAS-SF anxiety scores obtained immediately prior to mask induction. This primary outcome parameter of the study did not reveal any statistical significance between the ADV6209 group and the conventional midazolam group.

Table 3 gives an overview of the categorized treatment effects, showing trends both for higher numbers of positive and lower numbers of negative effects in the ADV6209 group. Both trends totaled up to an overhang of 10 children in whom ADV6209 had a positive effect or midazolam had a negative effect as compared to the other way around (i.e., either ADV6209 having a negative effect or midazolam having a positive effect).

Table 4 shows a significant difference in favor of ADV6209 with regard to patient’s oral acceptance of the premedication (*p* = 0.037). These were accepted by all children in the ADV6209 group, but rejected or spat out by 15% of children in the conventional midazolam group.

Table 5 summarizes the mask induction scores of how well the children accepted the facemasks placed for anesthesia induction, which did not show any statistically significant difference.

Hernia repairs, orchidopexies, cystoscopies, circumcisions and hypospadias corrections were the most frequently executed operations, whereas 23.8% of the patients were treated with balanced anesthesia (opioid and sevoflurane), 62.5% with regional anesthesia (neuroaxial/peripheral block) and sedation, 11.3% with a combined procedure (general anesthesia and regional anesthesia) and 2.5% with sedation alone.

## 4. Discussion

ADV6209 is a formulation of midazolam characterized by a γ-cyclodextrin molecular complex and labelled for oral premedication in pediatric anesthesia. The present study is the first clinical comparison between this novel substance and conventional midazolam, the most commonly used but off-label drug for oral premedication in children. Our comparative use of both variants in this randomized controlled and double-blinded study demonstrates on the basis of 80 ASA I/II patients 2−8 years old that ADV6209 is not superior to conventional midazolam in terms of preoperative anxiolysis. Both drugs revealed their efficacy in reducing anxiety and in providing adequate sedation. Nevertheless, ADV6209 was better accepted orally than conventional midazolam by children. Minimizing preoperative anxiety is an important goal in pediatric anesthesia, one that may even have implications for postoperative behavioral outcomes [15]. Psychological preparation [16,17,18] and pharmacological intervention [3,19] being the principal concepts involved, any steps of patient management in this context will aim for preoperative sedation and a smooth induction of anesthesia. Even though psychological concepts of minimizing preoperative anxiety in children have emerged as adequately effective from various studies [17], they are expensive to implement in daily clinical practice due to their time-consuming and labor-intensive nature. For the time being, pharmacological interventions will therefore continue to remain the standard approach to preoperative management in children. Given the need for preoperative drugs in pediatric anesthesia, these clinical situations are normally covered by oral (or sublingual, nasal,) or rectal applications that require no venous access [19], offer a fast pharmacological onset, and are free from side-effects such as respiratory depression, hypersalivation or paradox reactions. While many drugs have been explored in an effort to reduce preoperative anxiety, induce preoperative sedation and facilitate anesthesia induction, no such labelling was granted until recently. None of the major drug classes known to offer pertinent effects (benzodiazepines, NMDA-receptor antagonists, alpha-2-receptor agonists) meet all the requirements for preoperative pharmacological use in children, nor are they labelled for this specific indication [1,3,20].

ADV6209 is based on a new chemical formula which adds γ-cyclodextrin to midazolam, thereby improving solubility and palatability. Its development and formulation were first reported by Marçon and colleagues [5]. A pharmacodynamic and pharmacokinetic study by Guittet and colleagues has, more recently, yielded an insignificant effect of cyclodextrin on midazolam bioavailability [21]. In another study by Marçon and colleagues, ADV6209 revealed similar pharmacokinetics to extemporaneous oral solutions or syrups of midazolam, with no significant effects of cyclodextrin on clearance or central and peripheral volume distribution, and, confirming data from the literature, with a higher metabolic ratio and midazolam clearance per unit of body weight in the youngest patients [6].

While the European Union mandates approval of all drugs for all age groups, there is no denying that this goal has yet to be achieved [22,23,24,25,26]. Hence the continuing need for pediatric practitioners to administer drugs off-label does carry a risk of forensic implications. ADV6209 is a drug labelled for oral administration to achieve mild sedation in children. Approval has been carried on via a ‘decentralized procedure’ (DCP). The first licenses were granted in the United Kingdom and France in September 2018, and other European countries have since followed suit, including Austria in March 2020. Thus, ADV6209 is currently the only midazolam solution labelled for oral use in children, while the drug most widely used in clinical practice is the intravenous formulation of midazolam.

In this situation, it has been crucial to compare these two variants of oral premedication in a clinical study prior to establishing the new ADV6209 formulation routinely as the drug of choice. For most of the parameters evaluated in the present study, we obtained similar results with ADV6209 as with the conventional midazolam solution, but patient acceptance was significantly better. In particular, not a single child rejected or spat out an ADV6209 dose, whereas 15% of those receiving conventional midazolam did. It stands to reason that this better acceptance may also account for the other trends we noted in favor of ADV6209, namely, that anxiety levels (a) were lower at the time of mask induction and (b) had improved more often and deteriorated less often from baseline. One reason for obtaining trends in favor of ADV6209 but missing statistical significance may be to our power analysis. The assumed treatment effect was—retrospectively viewed—set to high. Furthermore, the power analysis was not based on a potential effect size affecting the oral acceptance score, which was one of our secondary outcome parameters.

On a closely related note, our study indicates that the orange taste of the ADV6209 formulation is well accepted by children. One might think of taste as an imponderable that could readily be factored out by using the nasal or rectal route of administration instead. However, Kogan and colleagues have shown that children accept the nasal route less favorably than the oral route [19], and our own departmental guidelines require us to avoid the rectal route in the age group of children investigated in this study (2–8 years). All this implies that oral administration is the preferred route of premedication and patient acceptance is an important consideration for its effect to be adequate. One limitation of our trial might be the fact, that our data are only generated for the mentioned age group (2–8 years). Certainly, the recruitment range could be widened in subsequent studies.

We included only children scheduled for anesthesia induction via facemask in this analysis, which could be considered as a limitation of the current study. This decision was based on the fact that anesthesia induction via facemask in children from 2–8 years represents our routine in the daily clinical practice, and we intended to avoid a heterogenous preanesthetic procedure. Nevertheless, the effect of ADV6209 on sedation levels during placement of an intravenous access needs to be investigated in a subsequent study.

## 5. Conclusions

In conclusion, ADV6209 is a sedative drug labelled for oral administration in children and better accepted than conventional midazolam by these patients. We also noted a trend for less anxiety at the time of anesthesia induction (30 min after the premedication). The latter finding arguably may be a consequence of the better oral acceptance, not least illustrated by zero cases of administered doses having been rejected or spat out in the ADV6209 group. Hence, ADV6209 is effective for its intended use and obviates the need for off-label drug use to premedicate children 2–8 years old.

## Figures and Tables

**Table 1:** Patient-related data.

	ADV6209		Midazolam		P
Age 2–4 years	17	(42.5%)	17	(42.5%)	1 *
Age 5–6 years	15	(37.5%)	15	(37.5%)	
Age 7–8 years	8	(20%)	8	(20%)	
ASA class I	36	(90%)	36	(90%)	1 †
ASA class II	4	(10%)	4	(10%)	
Female gender	12	(30%)	13	(32.5%)	1 †
Male gender	28	(70%)	27	(67.5%)	
Body weight, kg	20	(16–23)	20	(17–23.75)	0.56 ‡
Body height, cm	113	(104–122)	113	(104–123.5)	0.95 ‡

Absolute numbers with percentages or medians with interquartile ranges; * Pearson’s chi-square test; † Fisher’s exact test; ‡ Mann–Whitney U-test.

**Table 2:** Preoperative anxiety evaluated by mYPAS-SF* scores before and after premedication.

	ADV6209		Midazolam		P
Anxiety scores before premedication	29.16	(22.91–45.31)	28.12	(22.91–43.22)	0.57 †
Anxiety scores before mask induction	24.99	(22.91–36.97)	29.16	(22.91–39.58)	0.43 †

Medians and interquartile ranges; premedication was followed by mask induction after 30 min; * modified Yale Preoperative Anxiety Scale-Short Form; † Mann–Whitney U-test.

**Table 3:** Effects on mYPAS-SF* anxiety scores after as compared to before premedication.

	ADV6209		Midazolam		P
No effect (mYPAS-SF scores unchanged after 30 min)	16	(40%)	16	(40%)	0.348 †
Negative effect (mYPAS-SF scores higher after 30 min)	8	(20%)	13	(32.5%)	
Positive effect (mYPAS-SF scores lower after 30 min)	16	(40%)	11	(27.5%)	

Absolute numbers and percentages; premedication was followed by mask induction after 30 min; * modified Yale Preoperative Anxiety Scale-Short Form; † Pearson’s chi-square test.

**Table 4:** Acceptance of the premedication drug.

	ADV6209		Midazolam		P
Dose accepted readily	21	(52.5%)	23	(57.5%)	0.037 *
Dose accepted with a facial grimace	14	(35.0%)	9	(22.5%)	
Dose with accepted with vocal disapproval	5	(12.5%)	2	(5.0%)	
Dose rejected or spat out (almost) completely	0	(0.0%)	6	(15.0%)	

Absolute numbers (percentages); * Pearson’s chi-square test.

**Table 5:** Acceptance of facemask placed for inhalational anesthesia induction.

	ADV6209		Midazolam		P
Mask accepted well and immediately	29	(72.5%)	30	(75.0%)	0.62 *
Mask accepted after mild resistance	6	(15.0%)	5	(12.5%)	
Mask accepted after a moderate struggle	2	(5.0%)	4	(10.0%)	
Moderate force to overcome resistance	3	(7.5%)	1	(2.5%)	

Absolute numbers (percentages); * Pearson’s chi-square test.

## Data Availability

Data are contained within the article and Supplementary Material.

## Supplementary Materials

The following supporting information can be downloaded at: https://www.mdpi.com/article/10.3390/pharmaceutics14102062/s1, File S1: mYPAS-SF [8], Table S1: Parameters used for the power analysis, File S2: The CONSORT Checklist, File S3: The trial protocol [27,28,29,30,31,32].
