# Supplementary material for: ADV6209 for Premedication in Pediatric Anesthesia: A Double-Blinded, Randomized Controlled Trial
Source: Pharmaceutics. 2022 Sep 27;14(10):2062. doi: 10.3390/pharmaceutics14102062 (PMC9608962; doi:10.3390/pharmaceutics14102062)
Supplement: Supplementary file 1 [file pharmaceutics-14-02062-s001.zip › File S1.pdf]

File S1: The mYPAS-SF [8]

A. Activity

1 = Looking around, curious, playing with toys, reading (or other age-appropriate behavior); moves around holding area/treatment room to get toys or go to parent; may move toward OR equipment.

2 = Not exploring or playing, may look down, may fidget with hands or suck thumb (blanket); may sit close to parent while waiting, or play has a definite manic quality.

3 = Moving from toy to parent in unfocused manner, nonactivity-derived movements; frenetic/frenzied movement or play; squirming, moving on table, may push mask away, or clinging to parent.

4 = Actively trying to get away, pushes with feet and arms, may move whole body; in waiting room, running around unfocused, not looking at toys or will not separate from parent, desperate clinging.

B. Vocalizations

1 = Reading (nonvocalizing appropriate to activity), asking questions, making comments, babbling, laughing, readily answers questions but may be generally quiet; child too young to talk in social situations or too engrossed in play to respond.

2 = Responding to adults but whispers, "baby talk," only head nodding.

3 = Quiet, no sounds or responses to adults.

4 = Whimpering, moaning, groaning, silently crying.

5 = Crying or may be screaming "no."

6 = Crying, screaming loudly, sustained (audible through mask).

C. Emotional expressivity

1 = Manifestly happy, smiling, or concentrating on play.

2 = Neutral, no visible expression on face.

3 = Worried (sad) to frightened, sad, worried, or tearful eyes.

4 = Distressed, crying, extremely upset, may have wide eyes.

D. State of apparent arousal

1 = Alert, looks around occasionally, notices or watches what anesthesiologist does with him/her (could be relaxed).

2 = Withdrawn, child sitting still and quiet, may be sucking on thumb or face turned into adult.

3 = Vigilant, looking quickly all around, may startle to sounds, eyes wide, body tensed.

4 = Panicked whimpering, may be crying or pushing others away, turns away.

Scoring: Each item rating is divided by the highest possible rating (i.e., 6 for "vocalizations) and 4 for all other items), all produced values are added divided by 4, multiplied by 100.

22.9 = lowest scoring, 100 = highest scoring

8. Jenkins BN, Fortier MA, Kaplan SH, Mayes LC, Kain ZN. Development of a short version of the modified Yale Preoperative Anxiety Scale. Anesthesia and analgesia. 2014;119(3):643-50. Epub 2014/07/11. doi: 10.1213/ANE.0000000000000350. PubMed PMID: 25010821.
