# Supplementary material for: ADV6209 for Premedication in Pediatric Anesthesia: A Double-Blinded, Randomized Controlled Trial
Source: Pharmaceutics. 2022 Sep 27;14(10):2062. doi: 10.3390/pharmaceutics14102062 (PMC9608962; doi:10.3390/pharmaceutics14102062)
Supplement: Supplementary file 1 [file pharmaceutics-14-02062-s001.zip › File S3.pdf]

Medical University of Vienna
Department of Anaesthesia and Intensive Care Medicine
Spitalgasse23
A-1090 Vienna
Phone +43 1 40400 22330
Fax +43 1 40400 41280
Email: peter.marhofer@meduniwien.ac.at

THE USE OF ADV6209 FOR PREMEDICATION IN PAEDIATRIC ANAESTHESIA: A CONTROLLED, RANDOMIZED, DOUBLE BLINDED STUDY

CLINICAL STUDY PROTOCOL, Version 1.4

Date:Monday, 22. July 2019

Principal Investigator according to

Austrian drug law (AMG §§ 2a, 35, 36): Peter Marhofer, MD

Sponsor (AMG §§ 2a, 31, 32): Medical University of Vienna

Monitor (AMG §§ 2a, 33, 34) Edith Fleischmann, MD

Protocol Authors (in alphabetic order):
Peter **M**arhofer, MD
Philipp **O**pfermann, MD
Werner **S**chmid, MD
Lydia **T**riffterer, MD
Markus **Z**adrazil, MD

All: Department of Anaesthesia, Intensive Care Medicine and Pain Therapy, Medical University Vienna

Synopsis

TITLE	*The use of ADV6209 for premedication in paediatric anaesthesia:*<br>*A controlled, randomized, double blinded study*
Phase	IV
INVESTIGATIONAL PRODUCTS	**Drug:** ADV6209<br>***Dose:*** *0.25mg/kg*<br>**Drug:** Midazolam<br>***Dose:*** *0.25mg/kg*
BACKGROUND	To evaluate the effect of ADV6209, a new oral Midazolam formulation, on preoperative anxiety and sedation levels in paediatric anaesthesia
OBJECTIVES	**Primary Objective:** To compare the preoperative anxiety level using the anxiety score (mYPAS-SF) at induction of anaesthesia with face mask after premedication of the children with either ADV6209 (treatment group) or midazolam (control group).<br>**Secondary Objective:**<br>1) To compare the acceptance of anaesthesia mask at induction using the mask acceptance score after premedication of the children with either ADV6209 (treatment group) or midazolam (control group).<br>2) To compare the acceptance of the premedication drug - either ADV6209 (treatment group) or midazolam (control group) -after oral administration.
DESIGN	Prospective, controlled, randomized, double-blinded, single-centre
CENTER(S)<br>/ COUNTRY(IES)	1 centre in Austria (Department of Anaesthesia and Intensive Care Medicine, Medical University of Vienna)
PATIENTS / GROUPS	80 Children scheduled for elective surgery
INCLUSION CRITERIA	• ASA 1 and 2 children from 2-8 years scheduled for elective surgical or diagnostic procedures<br>• Signed written parental informed consent prior to inclusion in the study
EXCLUSION CRITERIA	• ASA 3-5

	• Allergy against the study drug<br>• Participation in another clinical study investigating another IMP within 1 month prior to screening<br>• Other objections to study participation in the opinion of the investigator
STUDY PROCEDURES	A screening visit precedes the first study day. For each individual, duration of the whole study will be approx. 45 min.<br>Once inclusion criteria are fulfilled and parental written informed consent to study participation is provided, participants will be randomized in one of two study groups.<br>Postinterventional final visit: At discharge to the ward
ADMINISTRATION OF STUDY DRUGS, STUDY GROUPS	**Study group 1:** ADV6209 0.25 mg/kg p.o.<br>**Study group 2:** Midazolam (in orange syrup) 0.25 mg/kg p.o.
ASSESSMENT OF CLINICAL EFFECT	mYPAS-SF<br>Face mask acceptance score<br>Study drug acceptance score
HOUSING	On the study day, subjects will be postoperatively transferred to the recovery room and discharged to the ward 2h later<br>For the whole duration of their stay, they will be under constant medical supervision.
STATISTICAL METHODOLOGY	Kurtosis and skewness of the mYPAS-SF will be assessed before definitive testing. If the pre-test indicated that data of mYPAS-SF was normally distributed a one-way analyses of variance will be performed. In case of non-normally distributed data the mYPAS-SF score at mask induction (= primary endpoint) will be compared Mann-Whitney U-test. We test the Hypothesis (H_0) that there is no difference in the mYPAS-SF score at mask induction after administration of either midazolam (control) or γ-cyclodextrin-Midazolam (treatment) for premedication.<br>The secondary endpoints namely a) the study drug acceptance score and b) the mask acceptance score will be compared using X^2 test as both scores are scaled in four categories. We test the Hypotheses (H_0) that there are neither significant differences in the

	study drug acceptance socre (a) nor in the mask acceptance score (b).<br><br>As we will do a stratified randomization (see above) a comparison of the study endpoints will be done within the strata (e.g.age and sex). To reduce the chances of obtaining false positive results (type I errors) when multiple pair wise tests are performed we use the Bonferroni correction for multiple comparisons.<br><br>To account for noncompliance, protocol deviations, withdrawal, and anything that happens after randomization the analysis will be done with an Intention-to-treat approach.<br><br>Results will be reported as mean ± SD or Median with IQR and/or percentage, if appropriate.<br><br>Differences were considered significant when $P < 0.05$. STATA12 (StataCorp, TX) and GraphPad Prism 5 (GraphPad, San Diego, CA) will be used for statistical analyses.

TABLE OF CONTENTS

1. SCIENTIFIC / MEDICAL SECTION 9

1.1 BACKGROUND 9
1.2 STUDY GROUPS 10
1.2.1 DOSING RATIONALE 10
1.2.2 ASSESSMENT OF OUTCOME VARIABLES 10
1.2.3 STORAGE OF STUDY DRUGS 11
1.3 OBJECTIVES OF THE STUDY 11
1.3.1 PRIMARY 11
1.3.2 SECONDARY 11
1.4 STUDY DESIGN 11

2. STUDY POPULATION 11

2.1 INCLUSION CRITERIA FOR STUDY SUBJECTS 11
2.2 EXCLUSION CRITERIA FOR STUDY SUBJECTS 12
2.3 REASON FOR WITHDRAWAL 12

3. STUDY DRUGS 12

3.1 ADV6209 12
3.1.1 PHARMACOKINETIC DATA 12
3.1.2 CONTRAINDICATIONS, UNDESIRABLE AND GENERAL SIDE EFFECTS 13
3.2 MIDAZOLAM 13
3.2.1 PHARMACOKINETIC DATA 13
3.2.2 CONTRAINDICATIONS, UNDESIRABLE AND GENERAL SIDE EFFECTS 13

4. STUDY PROTOCOL 14

4.1 SCREENING VISIT (DAY -14 TO DAY-1) 14
4.2 STUDY DAY 15
4.3 FINAL EXAMINATION 15

5. METHODS 15

5.1 ADMINISTRATION OF THE STUDY DRUG 15
5.2 MYPAS/MYPAS-SF 15
5.3 MASK ACCEPTANCE SCORE 17
5.4 ACCEPTANCE TO ADMINISTRATION OF THE STUDY DRUG 17
5.5 TIME POINTS 17

5.6 BLINDING 17

6. DRUG STORAGE 18

7. LABELING 18

7.1 SUBJECT IDENTIFICATION 18

8. LIFE STYLE RESTRICTIONS 18

9. DOCUMENTATION 18

10. ADVERSE EVENTS 20

10.1 DEFINITION 20
10.2 COMPLICATIONS OF PREMEDICATION 20
10.3 DOCUMENTATION AND REPORTING OF ADVERSE EVENTS 23
10.4 SERIOUS ADVERSE EVENTS (SAEs) 23
10.5 CAUSALITY ASSESSMENT BETWEEN ADVERSE EVENTS AND STUDY MEDICATION 23
10.6 SEVERITY OF THE ADVERSE EVENT 23
10.7 INFORMATION OF THE REGULATORY AUTHORITIES AND THE IRB-IEC 24

11. PREMATURE TERMINATION OF THE TRIAL 24

12. REMOVAL OF SUBJECT FROM TRIAL 24

13. STATISTICAL / ANALYSIS SECTION 25

13.1 RANDOMIZATION 25
13.2 STATISTICAL DATA ANALYSIS 25

14. ETHICAL SECTION 27

14.1 SAFETY PRECAUTIONS 27
14.2 INCONVENIENCES AND RISKS FOR SUBJECTS 27
14.3 RISK/BENEFIT ASSESSMENT 28
14.3.1 INDIVIDUAL BENEFIT 28
14.4 ADVERSE EVENTS 28
14.5 SERIOUS ADVERSE EVENTS 29

15. INSURANCE 30

16. ETHICAL AND LEGAL ASPECTS 30

17. AUDIT AND INSPECTION 31

18. ADMINISTRATIVE SECTION 31

18.1 PUBLICATION OF STUDY RESULTS 31
18.2 PROTOCOL AMENDMENT 31
18.3 MONITORING 31

19. CONFIDENTIALITY STATEMENT 31

20. FUNDING 32

21. APPENDICES 32

22. REFERENCES 34

GLOSSARY

AE	Adverse event
BMI	Body mass index
BP	Blood pressure
CRF	Case Report Form
ECG	Electrocardiogram
EOM	Extemporaneous Oral Solutions of Midazolam
EOS	End Of Study
EU	European Union
GCP	Good Clinical Practice
IMP	Investigational medicinal product
IRB	Institutional Review Board
i.v.	intravenous
mYPAS-SF	Modified Yale Preoperative Anxiety Scale - Short Form
OR	Operation Room
PD	Pharmacodynamic
PK	Pharmacokinetic
p.o.	per os
SAE	Serious Adverse Event
SUSAR	Suspected Unexpected Serious Adverse Reaction

1. SCIENTIFIC / MEDICAL SECTION

1.1 Background

Premedication represents an important part in the perioperative management of children and is used to reduce pre-operative stress as well as to facilitate induction of inhaled anaesthesia. Midazolam is the drug most often used for premedication in infants and children because it provides good sedation, anxiolytic and amnestic effects and few side effects [1]. Oral intake of the drug is the most preferred route of administration by paediatric patients[2], whereas the majority of existing oral Midazolam solutions involve the use of i.v. Midazolam formulation given orally, unregistered or off-label. Moreover i.v. Midazolam solutions are often not well accepted by children because of their bitter taste. Considering the lack of available oral medications for moderate sedation in children in most European countries, ADV6209, an innovative 0.2% (w/v) oral solution of Midazolam for children from six months of age, was developed. The formation of a γ-Cyclodextrin – Midazolam complex, representing a new chemical formula, was used to improve the solubility and the palatability of the Midazolam formulation[3]. A pharmacokinetic (PK) study has been executed in adults, children and adolescents, whereas ADV6209 showed improvements to current extemporaneous oral solutions of midazolam (EOM) preparations in terms of taste and easiness of use while presenting a similar PK profile[3]. Satisfying sedation, measured with the Observer Assessment of Alertness/Sedation Scale (OAA score / S≤17) was observed in 78.4% of paediatric patients 30 minutes after ADV6209 administration. Anxiety score (modified Yale Preoperative Anxiety Scale – mYPAS)[1] was decreased by 18.3% on average over baseline, 30 minutes after ADV6209 with a more pronounced effect in the youngest children who were also the most anxious prior to administration of the drug. In general the treatment was well accepted by all children and did not induce more crying and nausea / vomiting than before Midazolam[3]. ADV6209 seems a very promising premedication drug. It has been licensed by the EU regulatory authorities to become the first licensed paediatric sedative in the EU (September 2018). It may help to avoid unregistered and off label use of EOM. Consequently, we want to evaluate the efficacy and safety of ADV6209 on preoperative anxiety and sedation levels in paediatric anaesthesia in daily clinical routine in this randomized, controlled, double blinded study.

As ADV6209 is currently not available in Austria the study drug will be provided by the marketing authorisation holder Primex Pharmaceuticals AG, with the company headquarter in Obmoos 4, Ch-6300 Zug, Switzerland.

1.2 Study groups

In the present study, eighty ASA 1 and 2 children (age 2-8 years) will be included.

Study group 1: ADV6209 0.25mg/kg p.o.

Study group 2: EOM (Midazolam in syrup) 0.25mg/kg p.o.

1.2.1 Dosing rationale

An efficacy study of ADV6209 (Ozalin) has been executed, whereas sedation rates observed with ADV6209 were compared to increasing doses of oral Midazolam (Guittet et al. 2016). There was no significant difference between the response rates obtained with ADV6209 and those reported in historical studies (p=0.9428) at doses ranging from 0.2 to 1.5 mg/kg (no statistical difference between all doses was observed, p-value=0.2132). Whatever the model of dosage, the effect of the age group was not significant (p values>0.2). In addition a PK study has been performed where all subjects received a single 0.3 mg/kg dose of ADV6209, without exceeding a total dose of 10 mg (0.269 mg/kg on average)[3]. As there were no significantly better sedation rates with higher doses of Midazolam and the pediatric population deserves special precaution we decided for a dose of ADV6209 of 0.25mg/kg and similarly of 0.25mg/kg for midazolam. Moreover, according to the Summary of Product Characteristics of Ozalin (=ADV6209) the recommended dosage is 0.25mg/kg.

1.2.2 Assessment of outcome variables

The mYPAS has been used in several studies of diverse medical specialties, such as anaesthesia, surgery, pediatrics, and dentistry[4-7].The mYPAS proved to be an excellent tool for evaluating children's anxiety during the perioperative period. The mYPAS is of particular value to anaesthesiologists for evaluating new interventions directed toward reducing anxiety in children undergoing anaesthesia and surgery[1].

In 2014 Jenkins et. al proposed a short form of the modified mYPAS called the mYPAS-SF(Quelle). Compared to the original mYPAS score the elimination of two time points ("Walk to operating room" and „Entrance to operating room") and the exclusion of the „use of parents item" shortens the process of training raters to use the mYPAS-SF and this effectively reduces the time and effort in administration of the mYPAS (or mYPAS-SF) in half without any loss of significant clinical information regarding children's preoperative anxiety[8].

Two independent observers will evaluate the anxiety of the child (mYPAS-SF), the study drug acceptance score and the mask acceptance score. The observers will be trained.

In addition to this score we aim to rate the children's acceptance of the face mask and the acceptance of the oral administration of the premedication drug.

1.2.3 Storage of study drugs

The study drugs will be stored according to ICH-GCP. The sponsor determines, for the investigational products, acceptable storage temperatures, storage conditions (e.g. protection from light), storage times, reconstitution fluids and procedures, and devices for product infusion, if any. The sponsor informs all involved parties (e.g. monitors, investigators, nurses, storage managers) of these determinations. The investigational products will be packaged to prevent contamination and unacceptable deterioration during transport and storage.

1.3 Objectives of the study

1.3.1 Primary

Sedation score (mYPAS-SF) at induction of anaesthesia with the mask , i.e. 30 min after administration of the premedication drug)

1.3.2 Secondary

Acceptance of anaesthesia mask induction
Acceptance of oral administration of the premedication drug

1.4 Study design

Randomized, double blinded, controlled, single centre

Main activities during study period:

- Administration of the premedication drug ADV6209 vs. Midazolam
- Evaluation of the level of sedation using mYPAS-SF and a face mask acceptance score
- Evaluation of the level of study drug acceptance using a score

2. STUDY POPULATION

2.1 Inclusion criteria for study subjects

- ASA 1 and 2 children from 2-8 years scheduled for elective surgical or diagnostic procedures, where premedication would be used in clinical routine
- Signed written parental informed consent prior to inclusion in the study

2.2 Exclusion criteria for study subjects

- ASA 3-5
- Allergy against the study drug
- Participation in another clinical study investigating another IMP within one month prior to screening
- Other objections to study participation in the opinion of the investigator
- Parent's or legal guardian's refusal of participation of the child

2.3 Reason for withdrawal

Subjects may prematurely discontinue from the study at any time. Premature discontinuation from the study is to be understood when the subject did not undergo End of Study (EOS) examination and / or all pivotal assessments during the study.

Subjects must be withdrawn under the following circumstances:

- at their own request
- if the investigator feels it would not be in the best interest of the subject to continue
- if the subject violates conditions laid out in the consent form / information sheet or disregards instructions by the study personal

In all cases, the reason why subjects are withdrawn must be recorded in detail in the CRF and in the subject's medical records. Should the study be discontinued prematurely, all study materials (complete, partially completed and empty CRFs) will be retained.

3. STUDY DRUGS

3.1 ADV6209

3.1.1 Pharmacokinetic data

Formula: $C_{48}H_{80}O_{40}$- $C_{18}H_{13}ClFN_3$
Molecular mass: 1297.1248 g/mol - 325,77 g/mol
Bioavailability: 39.6% [8]
Protein binding: 94-96%
Metabolism: Midazolam is mainly eliminated by hydroxylation to form 1-Hydroxy-midazolam by CYP3A4 and CYP3A5 enzymes. This metabolite is glucuronidated before excretion into urine. Furthermore, there are two additional metabolites - the 4- Hydroxy-midazolam and the 1,4-Dihydroxymidazolam - emerging through glucuronidation. These two have minimal biological action and are excreted immediately into the urine.
Biological half-life: 3.6 ± 2.5h [8]
Excretion: Clearance of Midazolam is reduced in association with old age, congestive heart

failure, liver disease (cirrhosis) or conditions which diminish cardiac output and hepatic blood flow. The principal urinary excretion product is 1-Hydroxy-midazolam in the form of a glucuronide conjugate. Smaller amounts of the glucuronide conjugates of 4-Hydroxy- and dihydroxy-midazolam are also detected. The amount of midazolam excreted unchanged in the urine after a single i.v. dose is less than 0.5%.

3.1.2 Contraindications, undesirable and general side effects

As Midazolam is the active agent of ADV6209, the drug offers the same contraindications, undesirable and general side effects – see 3.2.2. and see Appendix 3

3.2 Midazolam

3.2.1 Pharmacokinetic data

Formula: $C_{18}H_{13}ClFN_3$=8-Chloro-6-(2-fluorophenyl)-1-methyl-4H-imidazol(1,5-α)(1,4)benzodiazepin
Molecular mass: 325,77 g/mol
Bioavailability: Bioavailability of intramuscular, rectal and „p.o." routes are 87, 18 and 27%.
Protein binding: 94-96%
Metabolism: Midazolam is mainly eliminated by hydroxylation to form 1-Hydroxymidazolam by CYP3A4 and CYP3A5 enzymes. This metabolite is glucuronidated before excretion into urine. Furthermore, there are two additional metabolites - the 4-Hydroxymidazolam and the 1,4-Dihydroxymidazolam - emerging through glucuronidation. These two have minimal biological action and are excreted immediately into the urine.
Biological half-life: 1,5-2,5h
Excretion: Clearance of Midazolam is reduced in association with old age, congestive heart failure, liver disease (cirrhosis) or conditions which diminish cardiac output and hepatic blood flow. The principal urinary excretion product is 1-Hydroxy-midazolam in the form of a glucuronide conjugate. Smaller amounts of the glucuronide conjugates of 4-Hydroxy- and Dihydroxymidazolam are also detected. The amount of Midazolam excreted unchanged in the urine after a single i.v. dose is less than 0.5%.

3.2.2 Contraindications, undesirable and general side effects

Contraindications: Hypersensitivity, severe respiratory disorder, severe liver insufficiency, Myasthenia gravis, sleep apnoea syndrome, closed angle glaucoma, chronic heart failure, constriction of blood vessels of the extremities, obstructive pulmonary disease, acute kidney failure, coma, shock, pregnancy

Undesirable and general side effects:

Hiccoughs, nausea, vomiting, coughing, "oversedation", headache, drowsiness, desaturation, apnea, hypotension, paradoxical reactions, seizure-like activity, nystagmus.

Other adverse experiences, observed mainly following i.v. injection as a single sedative/anxiolytic/amnesia agent and occurring at an incidence of < 1.0% in adult and pediatric patients, are as follows:

- Respiratory: Laryngospasm, bronchospasm, dyspnea, hyperventilation, wheezing, shallow respirations, airway obstruction, tachypnea.
- Cardiovascular: Bigeminus, premature ventricular contractions, vasovagal episode, bradycardia, tachycardia, nodal rhythm.
- Gastrointestinal: Acid taste, excessive salivation, retching.
- CNS/Neuromuscular: Retrograde amnesia, euphoria, hallucination, confusion, argumentativeness, nervousness, anxiety, grogginess, restlessness, emergence delirium or agitation, prolonged emergence from anaesthesia, dreaming during emergence, sleep disturbance, insomnia, nightmares, athetoid movements, seizure-like activity, ataxia, dizziness, dysphoria, slurred speech, dysphonia, paraesthesia.
- Special senses: Blurred vision, diplopia, nystagmus, pinpoint pupils, cyclic movements of eyelids, visual disturbance, difficulty focusing eyes, ears blocked, loss of balance, light-headedness.
- Integumentary: Hive-like elevation at injection site, swelling or feeling of burning, warmth or coldness at injection site.
- Hypersensitivity: Allergic reactions including anaphylactoid reactions, hives, rash, pruritus.
- Miscellaneous: Yawning, lethargy, chills, weakness, toothache, faint feeling, haematoma.

See Appendix 3

4. STUDY PROTOCOL

Upon signature of the informed consent form and provided that the inclusion/exclusion criteria are fulfilled, subjects will be enrolled in the study.

Overall duration of the study for each individual subject is planned to be approximately 45 min.

4.1 Screening visit (day -14 to day-1)

Prior to inclusion parents or legal guardians will be informed about the objective and the procedures of the study and about the risks involved. Inclusion and exclusion-criteria, demographic data including underlying disease will be assessed. Each enrolled child will be scheduled to undergo a general physical examination including anamnesis, and determination of body mass index (BMI).

4.2 Study day

All children are required to comply with the standard guidelines for preoperative fastening of our department (six hours for solid food, four hours for breast milk and one hour for clear fluids). According to randomisation the premedication is performed via the oral route with either ADV6209 0.25mg/kg or Midazolam in syrup 0.25mg/kg (each to a maximum dose 15 mg) thirty minutes prior induction of anaesthesia. When the child swallows the premedication drug the acceptance of oral administration of the respective drug is rated. The level of anxiety will be graded for each child using the mYPAS-SF performed at two different time points, which include preoperative holding before the premedication (time point t_1) and 30 min after premedication, when the anaesthesia mask is introduced to the child in the OR (t_2). After standard cardiorespiratory monitoring, induction of anaesthesia is performed via facemask and sevoflurane (initially 8 Vol %). The mask acceptance score will be applied instantaneously after mask induction. Standard monitoring includes ECG, noninvasive arterial blood pressure (BP), and peripheral oxygen saturation (SpO_2). The anaesthetic procedure will be executed as in daily clinical routine with no additional study related action. After the operation the child will be transferred to the recovery room and will be observed for 2h. Thereafter the child will be discharged to the ward.

4.3 Final examination

The final visit will take place, when the children are discharged from the postanaesthesia care unit to the ward.

5. METHODS

5.1 Administration of the study drug

Orange-flavoured γ-Cyclodextrin –Midazolam (ADV6209) (intervention group) or orange-flavoured Midazolam (control group) in syrup will be administered orally. For these purpose identically looking syringes for oral administration, in particular, containing either Midazolam (control group) or γ-Cyclodextrin –Midazolam (intervention group), will be prepared by an independent nurse and administered according to the allocation of the study participant. One member of the study team will mix Midazolam with the orange-flavoured syrup. The mixture is prepared adapted to the patients' bodyweight (0.25mg/kg Midazolam) in 5ml of orange-flavoured syrup. The nurse will fill the syringe with either the mixture of Midazolam in orange-flavoured syrup (control group) or with Ozalin (intervention group) to provide effective blinding.

5.2 mYPAS/mYPAS-SF

To enable the evaluation of child anxiety before surgery, the modified Yale Preoperative

Anxiety Scale (mYPAS) was developed in 1995[9] and modified in 1997[1](Appendix 1). The mYPAS has been used in several studies of diverse medical specialties, such as anaesthesia, surgery, pediatrics, and dentistry [4-7, 10, 11] .This scale uses five items, each representing a different field of child anxiety, and is mostly used at four points in time during the preoperative phase.

In 2014 Jenkins et. al proposed a short form of the modified mYPAS called the mYPAS-SF.Compared to the original mYPAS score the elimination of two time points (“Walk to operating room“ and „Entrance to operating room“) and the exclusion of the „parents item“ will shorten the process of training raters to use the mYPAS-SF and this effectively reduces the time and effort in administration of the mYPAS (or mYPAS-SF) in half without any loss of significant clinical information regarding children’s preoperative anxiety[8].

Content

The mYPAS-SF consists of four items (activity, vocalizations, emotional expressivity, state of apparent arousal). Each domain consists of Likert-type response options reflecting behaviours. Children’s behaviour is rated from 1 to 4 or 1 to 6 (depending on the domain), with higher numbers indicating the highest severity within that domain (see Appendix 1)[8].

Timing of assessment with mYPAS-SF

The mYPAS-SF is typically utilized at two different time points, which include preoperative holding and introduction to the anaesthesia mask[8].

Training

The four items of the mYPAS-SF are rated at each of the two time points by trained raters[8]. During training, raters first make themselves familiar with the mYPAS manual describing the purpose, administration, and scoring of the measure. Second, raters in training and previously trained raters evaluate videos as a group and review their scoring decisions. Finally, raters in training appraise videos alone and have their scores compared with previously trained raters’ scores[1]Any discrepancies between scores are assessed. Aim of the training is to achieve a high intra- and interrater reliability with Cohen’s *k* scores of at least 0.80.

Scoring

Ratings produce four mYPAS-SF scores (1 for each time point). Each score is calculated by

dividing each item rating by the highest possible rating (i.e., 6 for the “vocalizations” item and 4 for all other items), adding all the generated values, dividing by 4, and multiplying by 100Higher values indicate higher anxiety.

5.3 Mask acceptance score

A four-point mask-induction score will be used to determine the quality of induction of narcosis via face mask as follows: (1) Very good, immediate acceptance of the face mask, (2) good, slight resistance, (3) moderate, struggle against face mask and (4) difficult, moderate force necessary. See Appendix 2

5.4 Acceptance to administration of the study drug

Observers record the child’s acceptance of the orange-flavoured γ-Cyclodextrin –Midazolam (Ozalin®) (intervention group) or the Midazolam in orange - flavoured syrup (control group) according to the following graduation immediately after administration of the study drug: (1) The child accepts readily, (2) the child accepts with facial grimace, (3) the child accepts with verbal complaint or (4) the child rejects entirely or spits out almost/or all of the dose. See Appendix 2

5.5 Time points

In the holding area, when the child swallows the premedication drug, the acceptance of oral administration of the respective drug is rated. The level of anxiety will be graded for each child according to the mYPAS-SF at two different time points, which include preoperative holding (time point t_1) and 30 min after premedication, when the anaesthesia mask is introduced to the child in the OR (OR = time point t_2). The mask acceptance score will be applied instantaneously after mask induction.

5.6 Blinding

The study participants, healthcare providers (e.g. attending anaesthetists and surgeons) and data collectors are blinded for the allocation of the participant to the respective study group (intervention vs. control group). The similarity of the study drugs will be provided by similar taste (orange flavour), appearance (orange –colored) and viscosity of the administered drug. Also identical looking syringes for oral administration of the study drugs, in particular, containing either Midazolam (control group) in orange-flavoured syrup or γ-Cyclodextrin – Midazolam (intervention group), will be prepared. One member of the study team will mix Midazolam with the orange-flavoured syrup. According to randomization and to the exclusion of the investigators, an independent nurse will fill the syringe with either the mixture of Midazolam in orange-flavoured syrup (control group) or with Ozalin (intervention group) to provide effective blinding.

The mYPAS Score will be generated by two blinded raters, who assess independently of each other the anxiety status of the child. Also, two blinded assessors, who are not involved in the clinical care of the study participants, judge the “mask acceptance score” (s.a.) and the “acceptance of the orally administered drug” (s.a.) independently of each other.

6. DRUG STORAGE

As described above, the study drugs will be stored according to ICH-GCP. The sponsor determines, for the investigational products, acceptable storage temperatures, storage conditions (e.g. protection from light), storage times, reconstitution fluids and procedures, and devices for product infusion, if any. The sponsor informs all involved parties (e.g. monitors, investigators, nurses, storage managers) of these determinations. The investigational products will be packaged to prevent contamination and unacceptable deterioration during transport and storage. The identically looking syringes for oral administration of the study drugs containing either Midazolam (control group) or γ-Cyclodextrin–Midazolam (Ozalin®) (intervention group) are prepared by an independent nurse. One member of the study team will mix Midazolam with the orange-flavoured syrup to prepare the control product. After allocation the nurse will fill the syringe either with the mixture of Midazolam in orange-flavoured syrup (control group) or with Ozalin (intervention group).

7. LABELING

7.1 Subject identification

Subject numbers 01 - 80 will be used in a consecutive order. Subjects withdrawn from the study will retain their identification code. New replacement subjects enrolled will be assigned a number equal to the study number of the withdrawn subject plus 100 units: e.g. subject 01 will be replaced by the subject 101, subject 02 by subject 102.

8. LIFE STYLE RESTRICTIONS

The following restrictions will apply during the study: study participants should always be accompanied by a legal guardian.

9. DOCUMENTATION

The following documents must be available in the Investigator’s study file prior to the enrolment of any subject:

- Copy of the positive vote from the Ethics Committee;

- A list of members of the Ethics Committee in accordance with local regulations;
- Notification to local and national regulatory authority;
- Sample of the consent form and subject information to be used;

Data of screening examination will first be kept in separate subject files (source data files) and will be entered into the Case Report Form (CRF) only if the subject is eligible for trial participation and the data were verified by an investigator.

The subjects will be monitored through the course of the trial and all findings will be recorded.

The records should include the study number, the taking of consent, visit dates of the patient, medical history or examinations administered, laboratory results, concomitant treatment and sample collection times for diagnostic samples, any adverse events encountered, and other notes as appropriate.

AEs as reported by the patients will be recorded on separate forms as source data in German or English language and will be transcribed into the CRF after translation in English language. A physician will do the judgement according to the criteria of the ”Adverse Events” page.

Data of medical measurement without print-outs (vital signs, time of trial activities, i.e. time of sampling, administration) performed during the trial will be recorded on worksheets and will be handled as source data. Clinical laboratory parameters will be provided in laboratory print-outs which were signed and dated by the investigator if applicable. Comments on all clinically significant abnormal values should be given by the investigator. Data which are subject to the data protection law (i.e. name, address) will not be entered into the CRF.

For error corrections in source data the investigator or an authorized clinical staff member will cross out the part to be corrected (no correction fluid!) with one line, so that the incorrect data is still readable, and then entering the correct data, initialling and dating the change.

All correspondence (e.g. with Ethics Committee) relating to this clinical trial should be kept in appropriate file folders. Records of subjects, source documents, drug inventory sheet and Informed Consents pertaining to the trial must be kept on the Investigator’s File. Records must be retained according to guidelines governing the conduct of the trial.

According to the European Community record retention is required for 15 years.

If the investigator moves, withdraws from an investigation or retires, the responsibility for maintaining the records may be transferred to another person who will accept the responsibility.

10. ADVERSE EVENTS

10.1 Definition

An adverse event (AE) is any untoward medical occurrence in a subject or clinical investigation subject that does not necessarily have a causal relationship with any treatment. An adverse event may consist of any medical change (illness, condition, sign or symptom) in a subject after exposure to the study drug and includes:

- an event not seen prior to study drug administration
- an exacerbation of a pre-existing illness, condition, sign or symptom (e.g. increase in asthma attacks)
- a recurrence of an intermittent illness, condition, sign or symptom (e.g. migraine)
- an illness or condition diagnosed after study drug administration even though it may have been present prior to administration (e.g. cancer)

An adverse event does not include:

- a continuous persistent illness, condition, sign or symptom present before study drug administration which does not unexpectedly progress or change in severity following study drug administration
- the disease being studied or signs or symptoms associated with the disease (e.g., pain and swelling due to osteoarthritis of the knee)

10.2 Complications of Premedication

The following recommendations relate to the German prescribing information of Dormicum® (Midazolam) manufactured by Roche – see Appendix 3.

As any premedication drug, Midazolam should be administered only by experienced physicians in a setting fully equipped for the monitoring and support of respiratory and cardiovascular function and by persons specifically trained in the recognition and management of expected adverse events including respiratory and cardiac resuscitation.

Severe cardiorespiratory adverse events have been reported. These have included respiratory depression, apnoea, respiratory arrest and/or cardiac arrest. Such life-threatening incidents are more likely to occur when an i.v. injection is given and if the injection was too rapid or a high dosage was administered.

Special caution is required for the indication of conscious sedation in patients with impaired respiratory function.

Paediatric patients less than 6 months

In this population, Midazolam is indicated for sedation in ICU only. Paediatric patients less than 6 months of age are particularly vulnerable to airway obstruction and hypoventilation, therefore titration with small increments to clinical effect and careful respiratory rate and oxygen saturation monitoring are essential. When Midazolam is used for premedication, adequate observation of the patient after administration is mandatory as interindividual sensitivity varies and symptoms of overdose may occur. To avoid the potential risk for this vulnerable population we decided to include only children 2-8 years of age.

Special caution should be exercised when administering Midazolam to high-risk patients:

- adults over 60 years of age
- chronically ill or debilitated patients, e.g.
- patients with chronic respiratory insufficiency
- patients with chronic renal failure, impaired hepatic function or with impaired cardiac function
- paediatric patients specially those with cardiovascular instability.

These high-risk patients require lower dosages and should be continuously monitored for early signs of alterations of vital functions.

As with any substance with CNS depressant and/or muscle-relaxant properties, particular care should be taken when administering Midazolam to a patient with myasthenia gravis.

Tolerance

Some loss of efficacy has been reported when Midazolam was used as long-term sedation in intensive care units (ICU).

Dependence

When Midazolam is used in long-term sedation in ICU, it should be borne in mind that physical dependence on Midazolam may develop. The risk of dependence increases with dose and duration of treatment; it is also greater in patients with a medical history of alcohol and/or drug abuse . The issue of dependence is negligible in single use as premedication.

Withdrawal symptoms

During prolonged treatment with Midazolam in ICU, physical dependence may develop. Therefore, abrupt termination of the treatment will be accompanied by withdrawal symptoms. The following symptoms may occur: headaches, muscle pain, anxiety, tension, restlessness, confusion, irritability, rebound insomnia, mood changes, hallucinations and convulsions. Since the risk of withdrawal symptoms is greater after abrupt discontinuation of treatment, it is recommended to decrease doses gradually. The issue of withdrawal symptoms is negligible in single use as premedication.

Amnesia

Midazolam causes anterograde amnesia (frequently this effect is very desirable in situations such as before and during surgical and diagnostic procedures), the duration of which is directly related to the administered dose. Prolonged amnesia can present problems in outpatients, who are scheduled for discharge following intervention. After receiving Midazolam parenterally, patients should be discharged from hospital or consulting room only if accompanied by an attendant. Our study participants take the study drugs p.o. and are always accompanied by a legal guardian.

Paradoxical reactions

Paradoxical reactions such as agitation, involuntary movements (including tonic/clonic convulsions and muscle tremor), hyperactivity, hostility, rage reaction, aggressiveness, paroxysmal excitement and assault, have been reported to occur with Midazolam. These reactions may occur with high doses and/or when an i.v. injection is given rapidly. The highest incidence to such reactions has been reported among children and the elderly.

Altered elimination of Midazolam

Midazolam elimination may be altered in patients receiving compounds that inhibit or induce CYP3A4 and the dose of Midazolam may need to be adjusted accordingly.

Midazolam elimination may also be delayed in patients with liver dysfunction, low cardiac output and in neonates.

Preterm infants and neonates

Due to an increased risk of apnoea, extreme caution is advised when sedating preterm and former preterm non intubated patients. Careful monitoring of respiratory rate and oxygen saturation is required. Rapid injection should be avoided in the neonatal population. Neonates have reduced and/or immature organ function and are also vulnerable to profound and/or prolonged respiratory effects of Midazolam. Adverse haemodynamic events have been reported in paediatric patients with cardiovascular instability; rapid intravenous administration should be avoided in this population. In our trial Midazolam is administered p.o.

Concomitant use of alcohol / CNS depressants

The concomitant use of Midazolam with alcohol or/and CNS depressants should be avoided. Such concomitant use has the potential to increase the clinical effects of Midazolam possibly including severe sedation or clinically relevant respiratory depression.

Discharging criteria

After receiving Midazolam or other premedication drugs, patients should be discharged from hospital or consulting room only when recommended by treating physician and if accompanied by an attendant. It is recommended that the patient is accompanied when

returning home after discharge. Our study participants are always accompanied by a legal guardian.

10.3 Documentation and Reporting of Adverse Events

AEs will be documented throughout the entire study. In addition, SAEs which are reported to the investigator within 30 days after study termination have also to be documented using the SAE reporting form.

10.4 Serious adverse events (SAEs)

A serious adverse event is an adverse event that:

- results in death, or
- is life-threatening (the subject was at risk of death at the time of the event)
- requires in-patient hospitalisation or prolongation of existing hospitalisation
- results is persistent or significant disability / incapacity, or
- is a congenital anomaly / birth defect, or
- is another medically important condition that may be considered a SAE when, based upon appropriate medical judgment, it may jeopardize the subject or may require intervention to prevent one of the outcomes listed above

10.5 Causality Assessment between Adverse Events and study medication

A causality assessment between adverse events and study medication will be performed as follows:

Not related - clearly unrelated to the use of the test product; an evident alternative cause must be available.

Possible - causal relationship to the test product appears unlikely, but cannot be ruled out with reasonable certainty.

Probable - felt to be causally related to the test product with a high degree of likelihood.

10.6 Severity of the adverse event

The severity of AEs will be graded according to the following scale:

Mild - the adverse event does not interfere in a significant manner with the subject's normal functioning.

Moderate - the adverse event produces some impairment of functioning but is not hazardous to health.

Severe - the adverse event produces significant impairment of functioning or incapacitation and is a definite hazard to the subject's health.

10.7 Information of the regulatory authorities and the IRB-IEC

The sponsor has to inform the involved regulatory authorities and the investigator has to inform the involved ethics committee about each serious or unexpected AE/adverse drug reaction (that is an experience not previously reported - in nature, severity or incidence - in the current Summary of Product Characteristics that in the opinion of the investigator or sponsor might jeopardize the safety of the subjects or the further conduct of the study).

11. PREMATURE TERMINATION OF THE TRIAL

The trial will be terminated prematurely in any of the following cases for example:

- If adverse events occur which are so serious that the resulting risk-benefit ratio becomes unacceptable
- If the number of drop-outs is so large that proper conclusion of the trial is no longer a realistic possibility
- If the results of parallel clinical trials reveal unacceptable risks
- If results of any interim analyses and the status of drug development should change, such that the trial would no longer be a necessary part of the clinical program
- Attempted or proven fraud
- Slow recruitment
- Poor quality of data
- Non-compliance with protocol.

12. REMOVAL OF SUBJECT FROM TRIAL

Any subject who discontinues participation to the trial within two hours after onset of drug administration, is defined as "drop-out". Although subjects are not obliged to give reason(s) for withdrawal, the investigator should make a reasonable effort to ascertain the reason(s), while fully respecting the subject's rights.

The Investigator may remove the subject from the trial for any of the following reasons:

- Failure to observe the trial conditions or instructions from the trial team
- Failure to comply with any aspect of the protocol
- Occurrence of AEs

- Recurrent diseases which the investigator deems to be unacceptable
- Detection of drugs or addictive substances

For follow-up procedures of AEs the subjects will stay under medical supervision (in hospital or via telephone contact) unless the physician believes that all adverse events have resolved or a final assessment can be made. Appropriate control measurements may be initiated for safety parameters. All AEs will be documented and assessed by physicians. Serious adverse events will be communicated to the responsible authorities within the legal timelines.

If a subject is removed or discontinues, a replacement subject will be enrolled who will follow the same procedure as described for the original subject. The reason(s) for drop-out must be recorded.

13. STATISTICAL / ANALYSIS SECTION

13.1 Randomization

To ensure a balance between the treatment groups, we conduct a stratified randomization: Stratification will be done based on sex (female and male) and on three strata for age (2-4 years, 5-6 years, 7-8 years). Within each stratum (e.g. 2-4 years), half of the assignments are randomly selected to be to the control group and the other to be to the treatment group.

After inclusion of the patient and informed consent of the child´s parents or legal guardians a webbase randomization will be done (https://www.meduniwien.ac.at/randomizer/registered). The randomization process will be done by an independent person who is neither involved in the treatment nor in the investigation. Two sets (one main set, one backup set) of sealed envelopes with the randomization number containing information about treatment allocation will be prepared for each individual subject and kept throughout the study.

The treatment allocation (according to the randomization) will be confidential and will be known only for the independent nurseafter opening of the sealed envelope on the study day just before filling the syringe with either the mixture of Midazolam in orange-flavoured syrup (control group) or with Ozalin (intervention group) for oral premedication.

13.2 Statistical data analysis

In 2014 Jenkins et. al proposed a short form of the modified mYPAS called the mYPAS-SF(Quelle). Compared to the original mYPAS score the elimination of two time points (“Walk to operating room“ and „Entrance to operating room“) and the exclusion of the „parents item“ resulted in a change in mYPAS score from preoperative holding to introduction of the anesthesia mask produced effect sizes of 0.53 and 0.48 for the original mYPAS and the mYPAS-SF. The authors concluded that elimination of these items will shorten the process of

training raters to use the mYPAS-SF and this effectively reduces the time and effort in administration of the mYPAS (or mYPAS-SF) in half without any loss of significant clinical information regarding children's preoperative anxiety [8] .

To simplify the study process we therfore will rate the child's anxiety - contrary to what we planned first – with the mYPAS-SF at two timepoints: In the (1) holding area and at the (2) induction of the anaesthesia when the anaesthesia mask is introduced to the child in the OR.

Two independent observers will evaluate the child's anxiety (mYPAS-SF), the study drug acceptance socre and the mask acceptance score. The agreement between the two observers codifying the data will be verified through Cohen's *k* calculation[12].

The anxiety of the children in this study will be assessed using the mYPAS-SF, which is a continuous scale. Kurtosis and skewness of the mYPAS-SF will be assessed before definitive testing. If the pre-test indicated that data of mYPAS-SF was normally distributed a one-way analyses of variance will be performed. In case of non-normally distributed data the mYPAS-SF score at mask induction (= primary endpoint) will be compared Mann-Whitney U-test. We test the hypothesis (H_0) that there is no difference in the mYPAS-FS score at mask induction after administration of either midazolam (control) or γ-cyclodextrin-Midazolam (treatment) for premedication.

The secondary endpoints namely a) the study drug acceptance score and b) the mask acceptance score will be compared using X^2 test as both scores are scaled in four categories. We test the hypotheses (H_0) that there are neither significant differences in the study drug acceptance socre (a) nor in the mask acceptance score (b).

As we will do a stratified randomization (see above) a comparison of the study endpoints will be done within the strata (e.g.age and sex). To reduce the chances of obtaining false-positive results (type I errors) when multiple pair wise tests are performed we use the Bonferroni correction for multiple comparisons.

To account for noncompliance, protocol deviations, withdrawal, and anything that happens after randomization the analysis will be done with an Intention-to-treat approach.

Results will be reported as mean ± SD or Median with IQR and/or percentage, if appropriate.

Differences were considered significant when $P<0.05$. STATA12 (StataCorp, TX) and GraphPad Prism 5 (GraphPad, San Diego, CA) will be used for statistical analyses.

Data management:

Data were scrutinized for completeness, consistency, and outliers before analysis. In case of missing values, alternative data sources such as hospital records will be explored and missing data will be attempt to get completed by e.g. interviewing the child´s parents or by interrogation of the hospital database. Otherwise a conservative imputation strategy will be

considered. In such cases, values were replaced by appropriate subgroup mean or medians (eg, weight) as long as <10% were missing and only if patients with and without the respective information were similar with regard to other potential confounders.

Power analysis:

For the power analysis of this study we performed a literature research of Pub Med and Google Scolar to examined the effect sizes of mYPAS score differences within the literature. Studies that had both a control group receiving no treatment for preoperative anxiety and an intervention group receiving treatment for preoperative anxiety and additionally used the mYPAS as a measure of preoperative anxiety were use in this context. The effect sizes of Cohen *d* [12]in comparable studies dealing with midazolam premedication had a range from 0,48 to 0,65 suggesting a moderate treatment effect[13, 14]. The sample sizes described in these studies ranged from n= 50 to study n= 197 participants. As we compare the γ-cyclodextrin- midazolam (treatment group) with the midazolam (control group) for non-inferiority regarding the mYPAS-SF score 30 minutes after premedication power calculation was done assuming no or simply a small effect size of up to 0,2.

Based on this reflections we calcualted that group sample sizes of n = 25 (treatment group) and n=25 (control group) would achieve a 82% power to detect non-inferiority using a one-sided, two-sample t-test. The margin of equivalence was 10,000. The true difference between the means is assumed to be 0,000. The significance level (alpha) of the test was assumed to be 0,025. We calculated the power analysis with a dropout rate of 5%. This was done considering the rare event of cancelling an operation after the predication was already administered (e.g. technical problem with the OR, surgical emergency concerning another patient)

14. ETHICAL SECTION

14.1 Safety precautions

During the period of study visits, subjects will be observed by a physician or an experienced member of the nursing staff.

14.2 Inconveniences and risks for subjects

Side effects of ADV6209 or Midazolam (as enlisted in detail in the Summary of Product Characteristics, Appendix 3) include: Hiccoughs, nausea, vomiting, coughing, "oversedation", headache, drowsiness, desaturation, apnoea, hypotension, paradoxical reactions, seizure-like activity, nystagmus.

Other adverse experiences, observed mainly following i.v. injection as a single sedative/anxiolytic/amnesia agent and occurring at an incidence of < 1.0% in adult and paediatric patients, are as follows:

- Respiratory: Laryngospasm, bronchospasm, dyspnoea, hyperventilation, wheezing, shallow respirations, airway obstruction, tachypnoea.
- Cardiovascular: Bigeminus, premature ventricular contractions, vasovagal episode, bradycardia, tachycardia, nodal rhythm.
- Gastrointestinal: Acid taste, excessive salivation, retching.
- CNS/Neuromuscular: Retrograde amnesia, euphoria, hallucination, confusion, argumentativeness, nervousness, anxiety, grogginess, restlessness, emergence delirium or agitation, prolonged emergence from anaesthesia, dreaming during emergence, sleep disturbance, insomnia, nightmares, athetoid movements, seizure-like activity, ataxia, dizziness, dysphoria, slurred speech, dysphonia, paraesthesia.
- Special senses: Blurred vision, diplopia, nystagmus, pinpoint pupils, cyclic movements of eyelids, visual disturbance, difficulty focusing eyes, ears blocked, loss of balance, light-headedness.
- Integumentary: Hive-like elevation at injection site, swelling or feeling of burning, warmth or coldness at injection site.
- Hypersensitivity: Allergic reactions including anaphylactoid reactions, hives, rash, pruritus.
- Miscellaneous: Yawning, lethargy, chills, weakness, toothache, faint feeling, haematoma.

14.3 Risk/benefit assessment

Overall, the risks associated with the study are low. The risk-benefit ratio of the study is considered to be far on the side of the benefit due to the expected findings.

14.3.1 Individual benefit

Subjects have no additional individual benefit arising from the study as they are treated as in daily clinical routine. A general benefit of the trial would be the use of the only oral paediatric sedative that rapidly and consistently sedates children acceptably without bitterness of orally administered i.v. Midazolam formulations. In addition, the benefit is attributed to overall benefits for patients and scientific community, as ADV6209 has been licensed by the EU regulatory authorities since 2018 to become the first licensed paediatric sedative in the EU. This may induce avoiding unregistered and off label use and may change the way sedation is practised.

14.4 Adverse events

An adverse event (AE) is any untoward medical occurrence in a subject or clinical investigation subject that does not necessarily have a causal relationship with any treatment. An adverse event may consist of any medical change (illness, condition, sign or symptom) in a subject after exposure to the study drug and includes:

- an event not seen prior to study drug administration
- an exacerbation of a pre-existing illness, condition, sign or symptom (e.g. increase in asthma attacks)
- a recurrence of an intermittent illness, condition, sign or symptom (e.g. migraine)
- an illness or condition diagnosed after study drug administration even though it may have been present prior to administration (e.g. cancer)

An adverse event does not include:

- a continuous persistent illness, condition, sign or symptom present before study drug administration which does not unexpectedly progress or change in severity following study drug administration
- the disease being studied or signs or symptoms associated with the disease (e.g., pain and swelling due to osteoarthritis of the knee)

AEs will be documented throughout the entire study. In addition, SAEs which are reported to the investigator within 30 days after study termination have also to be documented using the SAE reporting form.

14.5 Serious adverse events

A serious adverse event is an adverse event that:

- results in death, or
- is life-threatening (the subject was at risk of death at the time of the event)
- requires in-patient hospitalisation or prolongation of existing hospitalisation
- results is persistent or significant disability / incapacity, or
- is a congenital anomaly / birth defect, or
- is another medically important condition that may be considered a SAE when, based upon appropriate medical judgment, it may jeopardize the subject or may require intervention to prevent one of the outcomes listed above

A causality assessment between adverse events and study medication will be performed as follows:

Not related - clearly unrelated to the use of the test product; an evident alternative cause must be available.

Possible - causal relationship to the test product appears unlikely, but cannot be ruled out with reasonable certainty.

Probable - felt to be causally related to the test product with a high degree of likelihood.

The severity of AEs will be graded according to the following scale:

Mild - the adverse event does not interfere in a significant manner with the subject’s normal functioning.

Moderate - the adverse event produces some impairment of functioning but is not hazardous to health.

Severe - the adverse event produces significant impairment of functioning or incapacitation and is a definite hazard to the subject’s health.

The sponsor has to inform the involved regulatory authorities and the investigator has to inform the involved ethics committee about each serious or unexpected AE/adverse drug reaction (SUSAR – Suspected Unexpected Serious Adverse Reaction, that is an experience not previously reported - in nature, severity or incidence - in the current Summary of Product Characteristics that in the opinion of the investigator or sponsor might jeopardize the safety of the subjects or the further conduct of the study). SUSARs not fatal or not life threatening will be reported to the IRB-IEC and the competent regulatory authorities within 15 calendar days. SUSARs that lead to death or are life threatening will be reported within 7 calendar days.

15. INSURANCE

For all study participants insurance is provided. Insurance is covered at the Zürich Insurance Group, Schwarzenbergplatz 15, A-1010 Vienna, telephone number 0800/0808080, with the policy number 07229622-2 and is valid from 01.07.2019 to 01.07.2020, 00.00 o’clock respectively. By request the parents or legal guardians can examine the insurance papers.

16. ETHICAL AND LEGAL ASPECTS

The study will be performed in accordance with the Declaration of Helsinki (1964), including current revisions, the Austrian Drug Law (Arzneimittelgesetz, AMG) (2004), Good Clinical Practice (GCP) guidelines of the European Commission and the Good scientific practice guidelines of the Medical University of Vienna. Approval from the local ethics committee will be sought before initiating the study. All subjects enrolled at the Department of Anaesthesia, Intensive Care Medicine and Pain Therapy, Medical University of Vienna, will be insured

through the Department of Anaesthesia, Intensive Care Medicine and Pain Therapy in accordance with §38 of the Austrian Medicines Act.

17. AUDIT AND INSPECTION

Upon request, the Investigator will make all study-related source data and records available to a qualified quality assurance auditor mandated by the competent authority inspectors. The main purposes of an audit or inspection are to confirm that the rights and welfare of the subjects have been adequately protected, and that all data relevant for assessment of safety and efficacy of the investigational product have appropriately been reported to the sponsor.

18. ADMINISTRATIVE SECTION

18.1 Publication of study results

Publication of study results from this investigation in an appropriate medical journal will be anticipated. Before submission the manuscript will be circulated to all parties involved.

18.2 Protocol amendment

If any modifications become necessary or desirable, these will be documented in writing; major changes require the approval of all investigators and the local ethics committee and competent authorities.

18.3 Monitoring

The Case Report Forms (CRFs) will be reviewed by the monitor. Also source data verification will be performed by the monitor to ensure accurate, consistent, reliable and complete data. Drug storage will be checked by the monitor. The study products should be dispensed by the investigator, or by a qualified individual under the investigator's supervision.

19. CONFIDENTIALITY STATEMENT

The information contained in this document is the property of the Department of Anaesthesia, Intensive Care Medicine and Pain Therapy, Medical University of Vienna and therefore is provided to you in confidence for review by you, your staff, an applicable Ethics Committee/Institutional Review and regulatory authorities. It is understood that the information will not be disclosed to others without prior written approval from the Department of Anaesthesia, Intensive Care Medicine and Pain Therapy, Medical University of Vienna except to the extent necessary to obtain informed consent from those persons to whom the medication may be administered.

20. FUNDING

No external funding will be used for this study.

21. APPENDICES

Appendix 1: The mYPAS-SF [8]

A. Activity
1 = Looking around, curious, playing with toys, reading (or other age-appropriate behavior); moves around holding area/treatment room to get toys or go to parent; may move toward OR equipment
2 = Not exploring or playing, may look down, may fidget with hands or suck thumb (blanket); may sit close to parent while waiting, or play has a definite manic quality
3 = Moving from toy to parent in unfocused manner, nonactivity-derived movements; frenetic/frenzied movement or play; squirming, moving on table, may push mask away or clinging to parent
4 = Actively trying to get away, pushes with feet and arms, may move whole body; in waiting room, running around unfocused, not looking at toys or will not separate from parent, desperate clinging

B. Vocalizations
1 = Reading (nonvocalizing appropriate to activity), asking questions, making comments, babbling, laughing, readily answers questions but may be generally quiet; child too young to talk in social situations or too engrossed in play to respond
2 = Responding to adults but whispers, "baby talk," only head nodding
3 = Quiet, no sounds or responses to adults
4 = Whimpering, moaning, groaning, silently crying
5 = Crying or may be screaming "no"
6 = Crying, screaming loudly, sustained (audible through mask)

C. Emotional expressivity
1 = Manifestly happy, smiling, or concentrating on play
2 = Neutral, no visible expression on face
3 = Worried (sad) to frightened, sad, worried, or tearful eyes
4 = Distressed, crying, extreme upset, may have wide eyes
D. State of apparent arousal
1 = Alert, looks around occasionally, notices/watches what anesthesiologist does with him/her (could be relaxed)
2 = Withdrawn, child sitting still and quiet, may be sucking on thumb or face turned into adult
3 = Vigilant, looking quickly all around, may startle to sounds, eyes wide, body tense
4 = Panicked whimpering, may be crying or pushing others away, turns away

Scoring: Divide each item rating by the highest possible rating (i.e., 6 for the "vocalizations" item and 4 for all other items), add all of the produced values, divide by 4, and multiply by 100.

Appendix 2: The acceptance scores:

The four-point mask-induction score:
(1) Very good, immediate acceptance of the face mask
(2) Good, slight resistance
(3) Moderate, struggle against face mask
(4) Difficult, moderate force necessary

The acceptance of oral administration score:
(1) The child accepts readily
(2) The child accepts with facial grimace
(3) The child accepts with verbal complaint
(4) The child rejects entirely or spits out almost/or all of the dose

Appendix 3: Prescribing information of Dormicum® - Midazolam injection solution, ROCHE

Summary of Product Characteristics of Ozalin

22. REFERENCES

1. Kain, Z.N., et al., *The Yale Preoperative Anxiety Scale: how does it compare with a "gold standard"?* Anesth Analg, 1997. **85**(4): p. 783-8.
2. Kain, Z.N., et al., *Trends in the practice of parental presence during induction of anesthesia and the use of preoperative sedative premedication in the United States, 1995-2002: results of a follow-up national survey.* Anesth Analg, 2004. **98**(5): p. 1252-9, table of contents.
3. Marcon, F., et al., *Development and formulation of a 0.2% oral solution of midazolam containing gamma-cyclodextrin.* Int J Pharm, 2009. **379**(2): p. 244-50.
4. Cuzzocrea, F., et al., *A psychological preoperative program: effects on anxiety and cooperative behaviors.* Paediatr Anaesth, 2013. **23**(2): p. 139-43.
5. Davidson, A.J., et al., *Risk factors for anxiety at induction of anesthesia in children: a prospective cohort study.* Paediatr Anaesth, 2006. **16**(9): p. 919-27.
6. Fortier, M.A., et al., *Perioperative anxiety in children.* Paediatr Anaesth, 2010. **20**(4): p. 318-22.
7. Fortier, M.A., et al., *Pediatric pain after ambulatory surgery: where's the medication?* Pediatrics, 2009. **124**(4): p. e588-95.
8. Jenkins, B.N., et al., *Development of a short version of the modified Yale Preoperative Anxiety Scale.* Anesth Analg, 2014. **119**(3): p. 643-50.
9. Kain ZN, M.L., Cicchetti DV *Measurement tool for preoperativeanxiety in young children: the Yale preoperatice anxiety scale.* Child Neuropsycho, 1995. **1**: p. 203-10.
10. Huet, A., et al., *Hypnosis and dental anesthesia in children: a prospective controlled study.* Int J Clin Exp Hypn, 2011. **59**(4): p. 424-40.
11. Weldon, B.C., M. Bell, and T. Craddock, *The effect of caudal analgesia on emergence agitation in children after sevoflurane versus halothane anesthesia.* Anesth Analg, 2004. **98**(2): p. 321-6, table of contents.
12. J, C., *Statistical Power Analysis for the Behavioral Sciences*. 2nd ed. Hillsdale, NJ: Lawrence Erlbaum Associates. 1988.
13. Kain, Z.N., et al., *Family-centered preparation for surgery improves perioperative outcomes in children: a randomized controlled trial.* Anesthesiology, 2007. **106**(1): p. 65-74.
14. Vagnoli, L., S. Caprilli, and A. Messeri, *Parental presence, clowns or sedative premedication to treat preoperative anxiety in children: what could be the most promising option?* Paediatr Anaesth, 2010. **20**(10): p. 937-43.
