# Supplementary material for: ADV6209 for Premedication in Pediatric Anesthesia: A Double-Blinded, Randomized Controlled Trial
Source: Pharmaceutics. 2022 Sep 27;14(10):2062. doi: 10.3390/pharmaceutics14102062 (PMC9608962; doi:10.3390/pharmaceutics14102062)
Supplement: Supplementary file 1 [file pharmaceutics-14-02062-s001.zip › Table S1.pdf]

Table S1: Parameters used for the power analysis.

We performed the POWER analysis:	*a priori*
on the primary outcome	mYPAS-SF anxiety score 30 minutes after the drug administration
based on the two-tailed statistical test	Two-tailed independent t-test
and accepting the cutoff for significance (α)	$P<0.05$
and a power (1-β) of	0.80
The variability of the primary outcome was:	Assumed with a standard deviation (SD) of 9 points for the mYPAS-SF 30 minutes after premedication
based on data taken from	Kain *et al*[13],<br>Vagnoli *et al*[14]
We considered as clinically relevant a difference of:	A decrease in the primary outcome measure from 36 in the control group to 30 mYPAS-SF points in the experimental group suggesting a moderate treatment effect (Cohen's D)
Consequently, the effect size was:	0.5
The total sample size needed was:	72 (36/group)
We calculated with a dropout rate of	2%
